# Molecular typing and virulence analysis of multidrug resistant *Klebsiella pneumoniae* clinical isolates recovered from Egyptian hospitals

**DOI:** 10.1038/srep38929

**Published:** 2016-12-22

**Authors:** Reham Wasfi, Walid F. Elkhatib, Hossam M. Ashour

**Affiliations:** 1 Department of Microbiology & Immunology, Faculty of Pharmacy, October University for Modern Sciences and Arts, Giza, Egypt; 2 Department of Microbiology & Immunology, Faculty of Pharmacy, Ain Shams University, Cairo, Egypt; 3 Department of Pharmacy Practice, School of Pharmacy, Chapman University, Orange, California, USA; 4 Department of Biological Sciences, College of Arts and Sciences, University of South Florida St. Petersburg, St. Petersburg, Florida, USA; 5 Department of Microbiology and Immunology, Faculty of Pharmacy, Cairo University, Cairo, Egypt

## Abstract

*Klebsiella pneumonia* infection rates have increased dramatically. Molecular typing and virulence analysis are powerful tools that can shed light on *Klebsiella pneumonia* infections. Whereas 77.7% (28/36) of clinical isolates indicated multidrug resistant (MDR) patterns, 50% (18/36) indicated carpabenem resistance. Gene prevalence for the AcrAB efflux pump (82.14%) was more than that of the mdtK efflux pump (32.14%) in the MDR isolates. *FimH-1* and *mrkD* genes were prevalent in wound and blood isolates. *FimH-1* gene was prevalent in sputum while *mrkD* gene was prevalent in urine. Serum resistance associated with outer membrane protein coding gene (*traT*) was found in all blood isolates. *IucC, entB,* and *Irp-1* were detected in 32.14%, 78.5% and 10.7% of MDR isolates, respectively. We used two Polymerase Chain Reaction (PCR) analyses: Enterobacterial Repetitive Intergenic Consensus (ERIC) and Random Amplified Polymorphic DNA (RAPD). ERIC-PCR revealed 21 and RAPD-PCR revealed 18 distinct patterns of isolates with similarity ≥80%. ERIC genotyping significantly correlated with resistance patterns and virulence determinants. RAPD genotyping significantly correlated with resistance patterns but not with virulence determinants. Both RAPD and ERIC genotyping methods had no correlation with the capsule types. These findings can help up better predict MDR *Klebsiella pneumoniae* outbreaks associated with specific genotyping patterns.

*K. pneumonia* belongs to family Enterobacteriaceaea and is related to other genera, such as *Enterobacter, Escherichia,* and *Salmonella*^1^. *K. pneumoniae* is considered one of the most common Gram negative bacteria^2^. It is also an important pathogen in nosocomial infections in Egypt^3^,^4^. A number of factors contribute to virulence and pathogenicity in *K. pneumoniae* such as the capsular serotype, lipopolysaccharide, iron-scavenging systems and adhesions^5^. Iron acquisition systems are essential for the growth of pathogenic bacteria^6^. Moreover, the iron chelator siderophore allows bacteria to take up protein-bound iron from the host cells^7^.

The incidence of microbial infections has been increasing in the past few decades. This has led to the continuous and uncontrolled use of antimicrobial drugs for prevention and treatment in several parts of the world. This, in turn, led to the emergence of specific drug and multidrug resistance among various strains of microorganisms including *K. pneumonia*^8^. Gram-negative bacteria have developed several mechanisms of resistance to currently used antimicrobials. One of the successful mechanisms for transmitting multiple-druge resistance among bacterial pathogens is horizontal transfer^9^. The spread of MDR isolates in the clinic has been attributed to commonly shared plasmids across bacteria such as *K. pneumoniae, K. oxytoca, Escherichia coli, Enterobacter sp.,* and *Salmonella sp*^10^,^11^. The efflux pump systems are among the most important causes of MDR^12^. Efflux pump systems in *K. pneumoniae* include AcrAB and mdtK systems, These belong to the Resistance Nodulation Division (RND) and Multi Antimicrobial Extrusion (MATE) family efflux pumps, respectively. The AcrAB-TolC pump is composed of an outer-membrane channel (TolC), a secondary transporter located in the inner membrane (AcrB), and a periplasmic component (AcrA)^13^. This pump is responsible for resistance to quinolones, tetracyclines, and chloramphenicol in various MDR isolates^14^. The MATE pumps, such as the mdtK system, transport some of those antimicrobial agents^15^. Porins such as OmpK35 and OmpK36 are crucial for the penetration of antibiotics into the cells and for susceptibility to cephalosporins and carbapenems^16^.

Carbapenems have been used for the treatment of infections caused by Enterobacteriaceae^17^. The percentage of Carbapenem-resistant Enterobactericeae (CRE) has been on the rise^18^. One of the most prominent recent increases of MDR was observed with *Klebsiella sp*. In the period from 2001 through 2011^18^. It is noteworthy that patients with infections due to carbapenemase-producing enterobacteriaceae, such as *K. pneumoniae* experience high mortality rates^19^,^20^,^21^. Normally, these MDR infections are hard-to-treat with limited available choices of antibiotics such as tigecycline, colistin, fosfomycin, and aminoglycosides^22^,^23^.

Molecular typing and virulence analysis of clinical isolates are powerful tools that can shed light on multidrug resistant (MDR) *Klebsiella pneumonia* infections. We also used two Polymerase Chain Reaction (PCR) genotyping analyses: Enterobacterial Repetitive Intergenic Consensus (ERIC) and Random Amplified Polymorphic DNA (RAPD) to assess correlations of each with resistance patterns, virulence determinants, or capsule types of *K. pneumoniae* isolates.

## Results

### Primers

Primers used for amplification are listed in Table 1. More detail is provided under materials and methods.

### Clinical isolates

Thirty six of *K. pneumoniae* clinical isolates were collected as described under materials and methods. Isolates were recovered from specimens of urine (n = 16), wound (n = 4), cerebrospinal fluid CSF (n = 1), blood (n = 7), sputum (n = 8) on MacConkey’s agar. Colonies showing lactose fermenting ability were further identified both microscopically and biochemically.

### Antimicrobial susceptibility pattern and detection of genes coding for MDR efflux pumps and outer membrane porins

As determined by disc diffusion antimicrobial susceptibility testing method, a percentage 77.7% (28/36) of isolates showed multidrug resistance (MDR) patterns, but all these MDR isolates were sensitive to colisitin (10 μg). All MDR isolates were resistant to beta lactam antibiotics and 64.28%, 82.15%, and 85.7% showed resistance to carbapenem, quinolone, and aminoglycosides, respectively. Tetracycline and chloramphenicol were effective against 61.1% of carbapenem-resistant isolates. The tested isolates were distributed into 24 antimicrobial resistance patterns (Table 2). Most patterns showed resistance to cephalosporin and beta lactam/beta lactamase inhibitors. The most predominant pattern was A6 and A8.

Gene prevalence for the AcrAB efflux pump system (82.14%) was more than that of the mdtK efflux pump (32.14%) in the MDR isolates. Incomplete AcrAB efflux pump system was detected in the remaining five isolates. The genes coding for porin protein (ompK35) and (ompK36) were not detected in six and four MDR isolates, respectively. Genes coding for the porins (*ompK 35* and *ompK36*) were detected in all isolates recovered from wound and CSF specimens. The presence of these two porin-coding genes was variable in blood, sputum, and urine samples.

### Detection of virulence genes

The prevalence and distribution of virulence factors are shown in Table 3. The *fimH-1* and *mrkD* genes, encoding type 1 and type 3 fimbrial adhesins, were present in all wound and blood isolates. The *fimH-1* gene was prevalent in sputum isolates whereas *mrkD* gene was prevalent in all urine samples. Serum resistance associated with the outer membrane protein coding gene (*traT*) was detected in all blood isolates.

The iron siderophores, aerobactin synthase gene (*IucC*), enterobactin biosynthesis gene (*entB*) and Yersinibactin biosynthesis gene (*Irp-1*) were detected in 32.14%, 85.7% and 28.5% of MDR isolates, respectively.

The prevalence of capsule K genotypes in the 28 MDR isolates revealed that K1 (n = 8), K2 (n = 2) and the remaining isolates were non-typable as K1 or K2 genotypes. Isolates showing K1 genotypes were obtained from urine, blood, and sputum specimens, while K2 isolates was recovered from urine and wound samples. There were 16 virulence profiles according to detected virulence genes. It is noteworthy that virulence genetic profiles indicated that virulence determinants were variable among *K. pneumoniae* strains that possess the same capsule genotype (Table 3).

### Genotyping of *Klebsiella pneumoniae* isolates by RAPD and ERIC analyses

**Figure 1:**
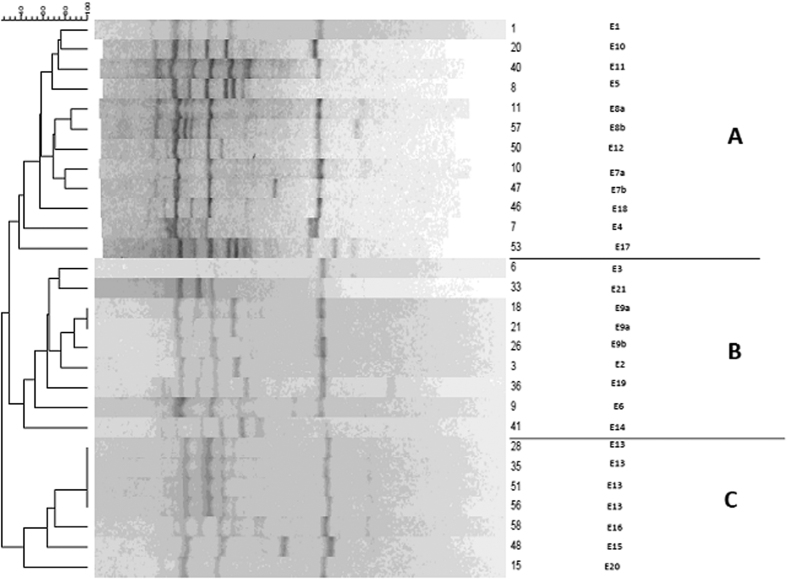
Dendrogram generated with Dice coefficient and the UPGMA clustering method, showing the genetic similarity among *K. pneumoniae* isolates by Enterobacterial Repetitive Intergenic Consensus (ERIC) genotyping.

**Figure 2:**
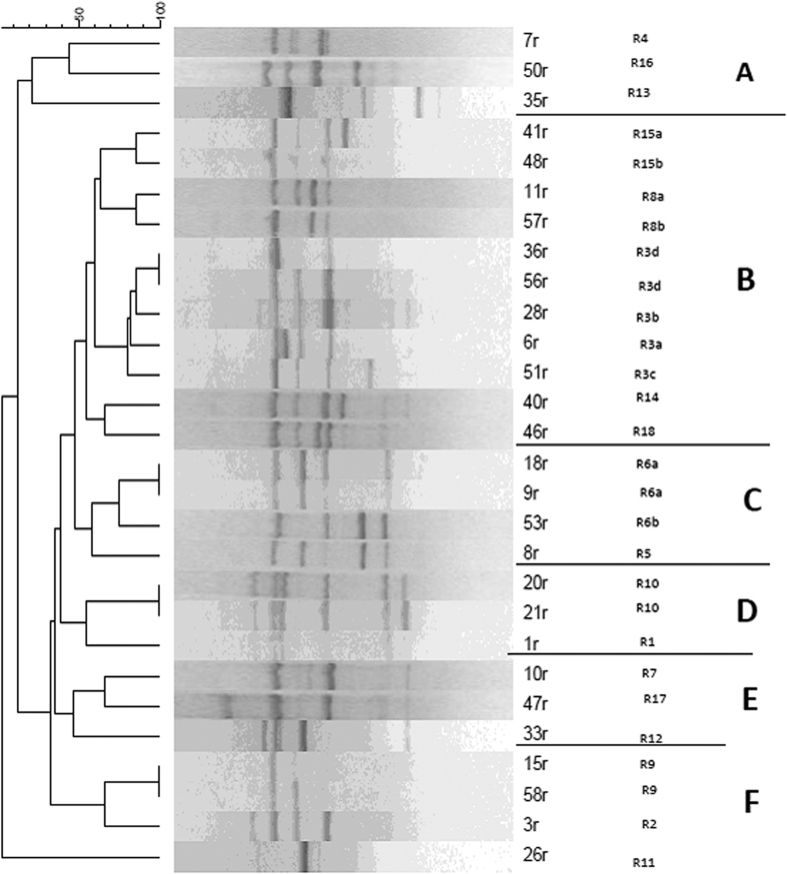
Dendrogram generated with Dice coefficient and the UPGMA clustering method, showing the genetic similarity among *K. pneumoniae* isolates by Random Amplified Polymorphic DNA (RAPD) genotyping.

According to the dendrograms, Enterobacterial Repetitive Intergenic Consensus (ERIC) and Random Amplified Polymorphic DNA (RAPD) analyses revealed 21 and 18 distinct patterns of *K. pneumoniae* isolates with similarity >80%, respectively (Figs 1 and 2). The 21 ERIC genotypes were designated E1 to E21 while the RAPD genotypes were designated R1 to R18 and each of their variant subtypes were indicated by a letter suffix. Dendrogram analysis of ERIC genotyping showed three clusters (A–C): clusters A, B, and C contained 12/28, 9/28, and 7/28 of the MDR isolates, respectively. The isolates (18 and 21) and (28, 35, 51, and 56) showed high similarity which may suggest that those isolates constitute a clonal lineage (Fig. 1). On the other hand, the RAPD genotyping revealed different pattern with 6 clusters (A–F). The isolates (15 and 58), (20 and 21), (9 and 18), and (36 and 56) showed high similarity (Fig. 2). Based on Simpson’s index of diversity, the discriminatory potential of different typing techniques used with *K. pneumoniae* isolates varied from 0.519 to 0.984 (Table 4). The high Simpson’s index of diversity for the antibiotyping, virulence, RAPD, and ERIC typing indicates greater diversity. Kendall’s tau-b correlation coefficient was calculated between RAPD and ERIC genotyping methods versus resistance patterns, virulence determinants, and capsule types of *K. pneumoniae*. Based on the statistical correlation tests (Table 5), the ERIC genotyping significantly correlates with resistance patterns (*p* < *0.01*) and virulence determinants (*p* < *0.05*). On the other hand, the RAPD genotyping significantly correlates with resistance patterns (*p* < *0.05*) but not with virulence determinants (*p* *>* *0.05*). Both RAPD and ERIC genotyping methods have no correlation with the capsule types (*p* *>* *0.05*).

## Discussion

*K. pneumoniae* is the causative agent of several different healthcare-associated infections, such as bloodstream infections, wound infections, pneumonia, and meningitis. The extensive use of antimicrobials led to high incidence of resistance in *K. pneumoniae*^24^. In our study, *K. pneumoniae* isolates showing multidrug resistance comprised 71.1% of total samples. Rates as high as 66.7% of MDR *K. pneumoniae* isolates were also detected in other studies^25^. The high rates of antimicrobial resistance detected in our study can be attributed to the lack of strict policies that govern the use of antibiotics in Egypt^3^.

Antibiotic efflux pumps represent one of the most important antimicrobial resistance mechanisms used by *K. pneumoniae* clinical isolates^26^,^27^. The increased efflux of the antimicrobial agent leads to the reduction of its intracellular concentration, which can enhance bacterial survival^28^. The AcrAB efflux pump was more common than mdtK. The presence of the multidrug efflux pump system (AcrAB-TolC) was significantly correlated with the MDR pattern. On the other hand, five MDR isolates was missing either the AcrAB efflux pump or the TolC outer membrane protein or both.

Gram negative bacterial outer membranes are poorly permeable to both hydrophobic and hydrophilic molecules. Thus, most antimicrobial agents other than β-lactam must cross the membrane in order to reach their intracellular drug targets and so require the presence of porin to bypass the asymmetric bilayer of phospholipid and lipopolysaccharide membrane^29^. Consequently, it has been reported that loss of porins ompK 35 and ompK 36 led to an increase in carbapenem, ciprofloxacin, and chloramphenicol resistance^30^. Surprisingly, in our research, porin loss was not significantly correlated to the MDR pattern (P > 0.05). This could be attributed to the presence of point mutations, disruption in the protein coding sequence, or promoter region mutations^31^.

In the current study, about fifty percent of the total isolates showed resistance to both imipenem and ertapenem. In this context, there has been a significant increase in carbapenem resistance among *K. pneumoniae* isolates in Egypt during the last few years (from 13.9% to 44.4%)^3^,^32^,^33^. *K. pneumoniae* isolates possess several mechanisms to evade the activity of carbapenems. These include AmpC production or ESBL production together with porin loss, carbapenemase production, and production of acquired Metallobetalactamase (MBL)^34^.

Contrary to the New Delhi metallo-β-lactamase, which is a broad spectrum carbapenemase with ability to inactivate β-lactams except aztreonam^35^, all carabapenem resistant isolates in our study were also resistant to aztreonem. This may be due to the development of a new antimicrobial resistance pattern in Egyptian hospitals. In all tested carbapenem-resistant isolates (n = 18), there was no simultaneous porin loss with AmpC or ESBL production. In another study^36^, Szabó *et al*. showed that OmpD and OmpF in an ertapenem-resistant *E. coli* strain were less permeable than those of a susceptible control strain. This suggested that the possession of these two porins could lead to higher resistance due to an associated pump system^36^. This is relevant to our study given the fact that OmpF genes in *E. coli* are homologues to OmpK35 genes in *K. pneumoniae*^29^.

Carbapenemase-producing enterobacteriaceae, such as *K. pneumonia*, can cause deadly infections^20^,^21^. Colistin was used to treat Gram-negative infections but was abandoned because of its toxicity. Recently, it has been revived again as a treatment for life-threatening infections caused by some resistant Gram-negative bacteria, such as *Pseudomonas aeruginosa* and *Acinetobacter baumannii*^37^. Interestingly, all the tested *K. pneumoniae* isolates in this study were sensitive to colistin.

Both OmpK35 and OmpK36 play a role in *K. pneumoniae* virulence and infection. Deletion of *OmpK36* or *OmpK35/OmpK36* can lead to the reduction in virulence of highly virulent strains and can increase their susceptibility to neutrophil phagocytosis^38^,^39^. In our investigation, both *Ompk 35* and *OmpK36* porin- coding genes were simultaneously detected in all *K. pneumoniae* isolates recovered from wound and CSF samples. Their presence was variable though in sputum, blood, and urine samples. A direct correlation between efflux pumps and virulence of pathogenic bacteria was reported by Padilla *et al*^40^. Several genes essential for intracellular invasion and survival were downregulated in mutant strains lacking *acrAB*-*tolC* efflux pumps^41^.

Type 1 fimbriae are the most common adhesive organelles in enterobacteriaceae and can lead to urinary tract infections^42^. Type 3 fimbrial adhesin can mediate the binding of *K. pneumoniae* to endothelial cells and to epithelial cells of the respiratory and urinary tracts. MrkD protein is a crucial factor in binding bacteria to the collagen molecules of the mammalian cells^43^. Many *K. pneumoniae* clinical isolated normally express both type 1 and type 3 fimbrial adhesins^44^. In the current study, the two genes coding for these adhesive structures (Type 3 fimbrial adhesin and MrkD) were detected in all wound, blood, and CSF isolates and in about 80% of sputum and urine isolates. The plasmidic *traT* gene encodes an outer membrane protein involved in bacterial conjugation and blocks the complement-mediated cascade, and act as an invasin^45^. We detected the *traT* gene in twenty two *K. pneumoniae* isolates (78.5%). The prevalence of *traT* gene in our isolates was relatively high as it was frequently associated with the K1 capsule serotype.

Most enterobacteriaceae strains contain genes encoding iron uptake systems, such as enterochelin or aerobactin^46^. These siderophores have dual roles as they can also inhibit T cell proliferation in addition to their role in enhancing iron uptake^47^. The iron siderophores aerobactin synthase gene (*IucC*), enterobactin biosynthesis gene (*entB*), and yersinibactin biosynthesis gene (*Irp-1*) were detected in 32.14%, 78.5%, and 10.7% of MDR *K. pneumoniae* isolates, respectively. Highly pathogenic Yersinia strains have high-pathogenicity island (HPI) that contain the gene *Irp-1*. This HPI is also prevalent in *Klebsiella* and other enterbocateria^48^, such as *E. coli, K. oxytoca, K. pneumonia, Citrobacter* species, and *Enterobacter* species^46^.

The capsular serotypes K1 and K2 are associated with the predominant virulent strains of *K. pneumoniae*^49^. Feizabadi *et al*. has shown that K1 and K2 serotypes represented 11.2% and 14.6%, respectively, of the total *K. pneumoniae* isolates^50^. In our study, K1 and K2 serotypes represented 28.5% and 7.14% of the MDR *K. pneumoniae* isolates.

Molecular typing is a potent tool for the study of nosocomial infections^51^. RAPD is a widely used genotyping tool for *K. pneumoniae* strains with ESBLs production (Gori *et al*., 1996). Out of a total of 28 MDR *K. pneumoniae* isolates in the current investigation, ERIC-PCR revealed 21 and RAPD-PCR revealed 18 distinct patterns of *K. pneumoniae* isolates. This may be attributed to the genetic variation in pathogenic these *K. pneumoniae* strains. Our data confirms the observations of Lai *et al*.^52^ that pathogenic *K. pneumoniae* is highly heterogeneous, due to differences in nucleotide sequences. The large number of serotypes in this species could also explain this genetic diversity highlighted by the RAPD-PCR genotypic analysis^53^.

Correlations between RAPD-PCR genotyping and antibiotic resistance patterns of *K. pneumonia* were observed by Ashayeri-Panah *et al*.^54^ and Espinar *et al*.^55^. In the current study, both RAPD-PCR and ERIC-PCR genotypic analyses revealed correlations with resistance patterns of *K. pneumonia*. The highest correlation coefficients were observed with ERIC genotyping, indicating that the latter may be more valuable in prediction of resistance patterns of *K. pneumoniae* as compared to the RAPD-PCR genotyping method. Moreover, ERIC, but not RAPD-PCR, revealed statistically significant correlations with virulence determinants of *K. pneumoniae*. Finally, both RAPD-PCR and ERIC-PCR showed no statistically significant correlation with the detected capsule types of *K. pneumoniae* isolates. Results included in this study can help up better predict MDR *Klebsiella pneumoniae* outbreaks associated with specific genotyping patterns in the future.

## Materials and Methods

### Bacterial strains

Thirty six *K. pneumonia* clinical isolates were recovered from patients at Kasr El Aini Hospitals, Cairo, Egypt. Approvals from the institutional review board of the hospitals and the Research Ethics committee of the October University for Modern Sciences and Arts, Giza, Egypt were obtained prior to conducting the study. All methods were performed in accordance with the required guidelines and regulations.

For experiments involving human samples, informed consent was obtained from all subjects. Strains were isolated from sputum, urine, blood, wound, and cerebrospinal fluid (CSF) specimens. Specimens were collected in the period from August 2015 through December 2015. Isolates were identified by conventional and biochemical tests as described previously^56^ and then were stored at −20 °C in brain heart infusion broth with 15% v/v glycerol.

### Antimicrobial susceptibility

Antibiotic susceptibility testing of *Klebsiella sp.,* was performed according to the Kirby-Bauer disk diffusion method^57^. The antimicrobial sensitivity assays to nineteen antibacterial drugs were done using commercially available antibiotic discs (OXOID, UK) including Ampicillin (AMP, 10 μg), Amoxicillin/Clavulanic acid (AMC, 20/10 μg), Piperacillin/Tazobactam (TZP, 110/10 μg), Cefoxitin (FOX, 30 μg), Ceftazidime (CAZ, 30 μg), Cefuroxime (CXM, 30 μg), Aztreonam (ATM, 30 μg), Ertapenem (ETP, 10 μg), Impinem (IMP, 10 μg), Gentamicin (CN, 10 μg), Tobramycin (TOB, 10 μg), Amikacin (AK, 30 μg), Tetracycline (TE, 30 μg), Doxycycline (DO, 30 μg), Ciprofloxacin (CIP, 5 μg), Nalidixic acid (NA, 30 g), Co-trimoxazole (SXT, 30 μg), Colistin (CT, 10 μg) and Chloramphenicol (C, 30 μg). For all tested antimicrobials except colistin (10 μg), the plates were then incubated at 37 °C for 24 hours, the diameters of the inhibition zones were measured in millimeter and interpretation of results was done according to CLSI standards^57^. For Colistin, breakpoints were used for interpretation^58^. Multidrug resistant (MDR) isolates were selected according to their non-susceptibility to at least one agent in three or more antimicrobial categories^59^.

### Detection of multidrug resistance and virulence determinants using PCR

#### DNA extraction

Genomic DNA was extracted from overnight culture using ZYMO Quick-gDNA™ MiniPrep (ZYMO Research, CA, USA). Concentration of the DNA extract and purity was determined by measuring absorbance at wavelengths 260 and 280 nm. The integrity of genomic DNA was tested by resolving DNA extracts on a 0.8% w/v agarose gel by electrophoresis. These crude DNA extracts were frozen at −20 °C.

Primers used for amplification are listed in Table 1 and were prepared by Invitrogen^®^ (Thermo Fisher scientific Inc., MA, USA). Primers were designed using the complete genome sequence of *K. pneumoniae* MGH 78578 (accession no. CP000647) and the internet based software Basic Local Alignment Tool (BLAST) in NCBI and Multiple sequence alignment using the CLUSTAL Omega in EMBL-EBI.

#### PCR detection of multidrug resistance genes

Isolates that showed multidrug resistance phenotypes were tested for genes coding for the multidrug efflux pump system *AcrAB-TolC* and *MdtK*, in addition to porin coding genes (*OmpK35* and *OmpK36*). The amplifications of these genes were performed in cycles with initial denaturing at 94 °C for 5 min followed by 35 cycles, each cycle consisting of 30 seconds at 94 °C for denaturation, 30 seconds for primer annealing (Table 1), and 1.5 min at 72 °C for elongation. After these cycles, the final elongation step was carried out at 72 °C for 10 min^45^.

#### PCR detection of virulence-associated genes

PCR was used to amplify the virulence-associated genes. These genes include those encoding for regulators of mucoid phenotype A (*rmpA*), type 1 and type 3 adhesins (*fimH-1, mrkD*), aerobactin (iron siderophore) synthase (*IucC*), bacteriocin biosynthesis [enterobactin (*entB*), and yersiniabactin (*irP-1*)], and serum resistance-associated outer membrane lipoprotein (*traT*). Measurements of the prevalence of capsule serotypes K1 and K2 were also included.

The PCR conditions were similar to those used for detection of multidrug resistance genes with annealing temperatures included in Table 1.

### Molecular typing of *Klebsiella pneumoniae* isolates using Random Amplified Polymorphic DNA (RAPD) and Enterobacterial Repetitive Intergenic Consensus (ERIC) methods

Typing by randomly amplified polymorphic DNA (RAPD) analysis was performed according to the protocol published by Deschaght *et al*.^60^ using the primer RAPD4 (5′-AAGACGCCGT-3′). Briefly, two microliters of the DNA template were added to 12.5 μL multiplex mastermix (MyTaq™HM Mix, Bioline^®^, MA, USA), 1 μL primer (10 pmol), and 9.5 μL H_2_O. PCR cycles of initial incubation at 94 °C for 15 min followed by cycling for 40 times at 94 °C for 1 min, 37 °C for 1 min, and a final elongation at 72 °C for 2 min was performed.

ERIC typing was carried out using the primer ERIC2 (5′-AAGTAAGTGACTGGGGTGAGCG-3′) using a similar PCR program to that of the RAPD method except for an extension time of 8 min^59^.

RAPD and ERIC fragments were visualized by 1.5% w/v agarose gel electrophoresis and results were analyzed using GelCompar II software (Version 6.6.11, Applied Maths, Kortrijk, Belgium). The patterns were normalized with bands of the marker and bands that were consistently present in all patterns. Computer-assisted analyses implemented in this study were performed according to the manufacturer’s instructions.

### Comparing different typing methods and calculation of discriminatory index

Simpsons index of diversity [discriminatory index (D)], based on the probability that two unrelated isolate samples from the test population are located in different typing groups, was calculated according to the following equation:


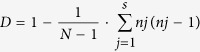


where N is the total number of isolates in the sample population, s is the total number of types described, and *nj* is the number of strains belonging to the *j*th type. Simpsons index of diversity ranges from 0.0 to 1.0, where 1.0 indicates that a typing method is able to distinguish each member of a population from all other members of that population and, conversely, 0.0 indicates that all members of a strain population are of an identical type^61^.

### Statistical analysis

All statistical analyses were performed using SPSS, version 18.0 (SPSS Inc., NY, USA). Chi-square tests were used to compare categorical measures between groups (Fisher’s exact test where appropriate). Statistical correlation tests, including Kendall’s tau-b nonparametric correlation coefficients, were determined at the two-tailed significance level for correlation of genotyping methods with virulence determinants, antimicrobial resistance, and capsule types. Data output of correlation analyses with p values less than 0.05 were considered statistically significant.

## Additional Information

**How to cite this article**: Wasfi, R. *et al*. Molecular typing and virulence analysis of multidrug resistant *Klebsiella pneumoniae* clinical isolates recovered from Egyptian hospitals. *Sci. Rep.* **6**, 38929; doi: 10.1038/srep38929 (2016).

**Publisher's note:** Springer Nature remains neutral with regard to jurisdictional claims in published maps and institutional affiliations.

## Figures and Tables

**Table 1:** List of primers, expected amplicon size, and annealing temperatures.

Gene	Primer Sequence (5′-----3′)	Amplicon size (bp)	Tm °C	Reference
*RmpA*	For: ACTGGGCTACCTCTGCTTCARev: CTTGCATGAGCCATCTTTCA	535	53	Siu, *et al*.^62^
*fimH-1*	For: GCCAACGTCTACGTTAACCTGRev: ATATTTCACGGTGCCTGAAAA	180	43	The current study
*mrkD*	For: CCACCAACTATTCCCTCGAARev: ATGGAACCCACATCGACATT	226	43	El Fertas-Aissani, *et al*.^45^
*arb*	For: TGGGGCAAAGAGGCGCTG GAGRev: CAGCCAGCGACACGGATTCTC	636	51	The current study
*entB*	For: CTGCTGGGAAAAGCGATTGTCRev: AAGGCGACTCAGGAGTGGCTT	385	49	The current study
*irP-1*	For: TGAATCGCGGGTGTCTTATGCRev: TCCCTCAATAAAGCCCACGCT	238	49	El Fertas-Aissani, *et al*.^45^
*traT*	For: GGTGTGGTGCGATGAGCACAGRev: CACGGTTCAGCCATCCCTGAG	288	55	El Fertas-Aissani, *et al*.^45^
*AcrAB*	For: ATCAGCGGCCGGATTGGTAAARev: CGGGTTCGGGAAAATAGCGCG	312	53	The current study
*tolC*	For: ATCAGCAACCCCGATCTGCGTRev: CCGGTGACTTGACGCAGTCCT	527	51	The current study
*mdtk*	For: GCGCTTAACTTCAGCTCARev: GATGATAAATCCACACCAGAA	453	43	The current study
*OmpK35*	For: CTCCAGCTCTAACCGTAGCGRev: GGTCTGTACGTAGCCGATGG	241	51	The current study
*OmpK36*	For: GAAATTTATAACAAAGACGGCRev: GACGTTACGTCGTATACTACG	305	43	The current study
*K1*	For: GGTGCTCTTTACATCATTGCRev: GCAATGGCCATTTGCGTTAG	1283	47	Fang, *et al*.^63^
*K2*	For: GGATTATGACAGCCTCTCCTRev: CGACTTGGTCCCAACAGTTT	908	45	Fang, *et al*.^63^

**Table 2:** Antimicrobial sensitivity patterns of multidrug resistant *Klebsiella pneumoniae* isolates and prevalence of genes coding for MDR efflux pumps (*AcrAB* & *MdtK*) and outer membrane porins (OmpK35 & OmpK36).

Anti-biotype	Isolate No.	Antimicrobial resistance profile	Genes coding for porins and efflux pumps				
			*OmpK35*	*OmpK36*	*MdtK*	*TolC*	*AcrAB*
A1	1w	AMP-AMC-TZP-FOX-CAZ-CXM-AZM-IMP-ETP-AMK^∆^-CN-TOB-CIP-NA-C	+	+	+	+	+
A2	3 s	AMP-AMC- CAZ-CXM-AZM-TE-DOX^∆^-NA^∆^-SXT	−	−	−	+	+
A3	6w	AMP-AMC-TZP-FOX^∆^-CAZ-CXM-AZM-CN-TOB-CIP-NA^∆^-SXT-	+	+	−	+	+
A4	7 s	AMP-AMC-TZP-FOX-CAZ-CXM-AZM-IMP-ETP-CN-TOB	−	−	−	+	+
A5	8 s	AMP-CAZ-CXM-AZM-TOB-TE-SXT-C	−	+	−	+	+
A6	9 u	AMP-AMC-TZP-FOX-CAZ-CXM- IMP-ETP-AK-CN-TOB-CIP-NA	+	+	−	+	+
	21c		+	+	+	+	+
	26w		+	+	+	+	−
A7	10 u	AMP-AMC-TZP-FOX-CAZ-CXM-AZM-IMP-ETP-AK-TOB-CIP-NA-SXT-C^∆^	+	+	+	+	+
A8	11 u	AMP-AMC-TZP-FOX-CAZ-CXM-AZM-IMP-ETP-AK-CN-TOB-TET-DOX-SXT-CIP-NA-C	−	+	+	+	+
	40b		+	−	−	+	+
	48b		−	+	−	+	+
A9	15 u	AMP-AMC-TZP-FOX-CAZ-CXM-AZM-IMP-ETP-AK-CN-TOB- SXT-CIP-NA-C	+	+	−	+	+
A10	18 u	AMP-AMC-CAZ-CXM-AZM- TET-DO-SXT- C	−	+	−	+	+
A11	20 s	AMP-AMC-TZP-FOX-CAZ-CXM-AZM-IMP-ETP-AK-CN-TOB- DO-SXT-CIP-NA-C	+	+	+	−	+
A12	28 s	AMP-AMC-TZP-FOX^∆^-CAZ-CXM-AZM-AK- ETP^∆^-TET-DO^∆^-SXT-CIP-NA	+	+	−	+	+
A13	33 u	AMP-AMC-TZP-FOX-CAZ-CXM-IMP-ETP-AK-CN-TOB- CIP-NA	+	+	−	−	+
A14	35b	AMP-AMC-TZP-FOX-CAZ-CXM-IMP-ETP-AK-CN-TOB- DO-TET^∆^-SXT-CIP-NA	+	+	+	+	+
A15	36 u	AMP-AMC-TZP- CAZ-CXM-AZM-CN-TOB-TET-AK-DO-SXT^∆^-CIP-NA	+	+	−	+	+
A16	41b	AMP-AMC-TZP-FOX-CAZ-CXM-IMP-ETP-AK- TOB-CIP^∆^	+	−	−	+	+
A17	46w	AMP-AMC-CAZ-CXM- CN-TOB-TET-DOX^∆^-CIP^∆^- SXT	+	+	+	−	+
A18	47b	AMP-AMC-TZP-FOX-CAZ-CXM-AZM-IMP-ETP- AK^∆^-CN-TOB-TET-DO- CIP-NA-SXT^∆^	+	+	+	+	+
A19	50 u	AMP-AMC-TZP-FOX-CAZ-CXM-AZM - CN- DO-SXT-CIP^∆^-NA	+	+	−	+	+
A20	51 u	AMP-AMC-TZP-FOX-CAZ-CXM-AZM-IMP-ETP-CN-TOB-AK^∆^-CIP-NA^∆^-SXT	+	−	−	+	+
A21	53 u	AMP-AMC-TZP^∆^-CXM^∆^-AZM^∆^- CN-TOB-TET-DO-SXT-CIP-NA-C	+	+	−	−	+
A22	56 u	AMP-AMC-TZP-FOX-CAZ-CXM-AZM-IMP-ETP-CN-TOB-AMK-TET-DO-CIP-NA-SXT	+	+	−	+	+
A23	57 u	AMP-AMC-TZP-FOX-CAZ-CXM-CN-TOB^∆^- TET-SXT-CIP-NA-C	+	+	−	+	+
A24	58 u	AMP-AMC-TZP-FOX-CAZ-CXM-AZM-SXT-NA^∆^- C	+	+	−	+	+

Abbreviations: u: urine, w- wound, s: sputum, c: CSF, b: blood.

^∆^Intermediate sensitivity.

AMP: ampicillin; AMC: Amoxicillin/clavulanic acid; TZP: Piperacillin/tazobactam; CAZ: Ceftazidime; CXM: Cefuroxime; FOX: Cefoxitin; AZM: Azteronam; IMP: Imipenem; ETP: Ertapenem; AK: Amikacin; TET: Tetracycline; DO: Doxycycline; CN: Gentamicin; TOB: Tobramycin; CIP: Ciprofloxacin; NA: Nalidixic acid; SXT: Co-trimoxazole; C: Chloramphenicol.

**Table 3:** Distribution of virulence genetic profiles of *K. pneumoniae* isolates among capsule genotypes.

Isolate code*	Capsule serotype	*Virulence gene*							Virulence genetic profile
		*rmpA*	*fimH-1*	*mrkD*	*traT*	*entB*	*Irp-1*	*IucC*	
1w	Non K1/K2	+	+	+	+	+	+	+	V1
3s	K1	−	+	+	+	−	+	−	V2
6w	K2	−	+	+	−	+	−	+	V3
7s	Non K1/K2	−	+	+	−	+	−	+	V4
8s	K1	−	+	+	+	+	+	−	V5
10u									
41b									
48b									
53u									
9u	K2	−	+	+	+	+	−	−	V6
11u	K1	+	+	+	+	+	−	+	V7
15u	Non K1/K2	−	+	+	−	+	−	−	V8
20s									
46w									
18u	Non K1/K2	−	+	+	+	+	−	−	V9
26w									
35b									
56u									
58u									
21c	Non K1/K2	+	+	+	+	+	−	−	V10
50u									
57u									
28s	Non K1/K2	−	+	−	+	+	−	+	V11
33u	Non K1/K2	−	−	+	+	−	−	−	V12
36u	Non K1/K2	−	−	+	+	+	−	+	V13
40b	K1	−	+	+	+	−	−	+	V14
47b	Non K1/K2	−	+	+	+	+	+	+	V15
51u	Non K1/K2	−	+	+	+	+	−	+	V16

Abbreviations: u: urine, w- wound, s: sputum, c: CSF, b: blood. Non K1/K2 = Non typable as K1 or K2 capsule genotypes.

**Table 4:** Discriminatory potential of typing techniques for *K. pneumoniae* isolates.

Isolate code	Antibiogram	Virulence gene pattern typing	Capsule serotyping	ERIC typing	RAPD typing
1w	A1	V1	Non K1/K2	E1	R1
3s	A2	V2	K1	E2	R2
6w	A3	V3	K2	E3	R3a
7s	A4	V4	Non K1/K2	E4	R4
8s	A5	V5	K1	E5	R5
9u	A6	V6	K2	E6	R6a
10u	A7	V5	K1	E7a	R7
11u	A8	V7	K1	E8a	R8a
15u	A9	V8	Non K1/K2	E20	R9
18u	A10	V9	Non K1/K2	E9a	R6a
20s	A11	V8	Non K1/K2	E10	R10
21c	A6	V10	Non K1/K2	E9a	R10
26w	A6	V9	Non K1/K2	E9b	R11
28s	A12	V11	Non K1/K2	E13	R3b
33u	A13	V12	Non K1/K2	E21	R12
35b	A14	V9	Non K1/K2	E13	R13
36u	A15	V13	Non K1/K2	E19	R3d
40b	A8	V14	Non K1/K2	E11	R14
41b	A16	V5	K1	E14	R15a
46w	A17	V8	Non K1/K2	E18	R18
47b	A18	V13	Non K1/K2	E7b	R17
48b	A8	V5	K1	E15	R15b
50u	A19	V10	Non K1/K2	E12	R16
51u	A20	V15	Non K1/K2	E13	R3c
53u	A21	V5	K1	E17	R6b
56u	A22	V9	Non K1/K2	E13	R3d
57u	A23	V10	Non K1/K2	E8b	R8b
58u	A24	V9	Non K1/K2	E16	R9
Simpsons index of diversity	0.984	0.925	0.519	0.969	0.955

**Table 5:** Kendall’s tau-b correlation coefficient of Random Amplified Polymorphic DNA (RAPD) and Enterobacterial Repetitive Intergenic Consensus (ERIC) genotyping methods versus resistance patterns, virulence determinants, and capsule types of *K. pneumoniae* isolates.

Genotyping method	Resistance pattern	Virulence determinant	Capsule type
RAPD	0.306* (*p* < *0.05*)	0.268 (*p* = *0.053*)	0.106 (*p* = *0.497*)
ERIC	0. 520* (*p* < *0.01*)	0.352* (*p* < *0.05*)	0.210 (*p* = *0.181*)

^*^Correlation is significant at *p* < *0.05* (two-tailed).
